# Molecular drivers of resistance to sulbactam-durlobactam in contemporary clinical isolates of *Acinetobacter baumannii*

**DOI:** 10.1128/aac.00665-23

**Published:** 2023-10-16

**Authors:** Samir H. Moussa, Adam B. Shapiro, Sarah M. McLeod, Ramkumar Iyer, Nicole M. Carter, Yu-Kuo Tsai, L. Kristopher Siu, Alita A. Miller

**Affiliations:** 1 Innoviva Specialty Therapeutics Inc., an affiliate of Entasis Therapeutics Inc., Waltham, Massachusetts, USA; 2 Kemyth Biotech Co., Ltd., Taipei City, Taiwan; University of Fribourg, Fribourg, Switzerland

**Keywords:** *Acinetobacter*, sulbactam, durlobactam, surveillance studies, resistance, ETX2514

## Abstract

*Acinetobacter baumannii*-*calcoaceticus* complex (ABC) causes severe infections that are difficult to treat due to pre-existing antibiotic resistance. Sulbactam-durlobactam (SUL-DUR) is a targeted β-lactam/β-lactamase inhibitor combination antibiotic designed to treat serious infections caused by *Acinetobacter*, including multidrug- and carbapenem-resistant strains. In a recent global surveillance study of 5,032 ABC clinical isolates collected from 2016 to 2021, less than 2% of ABC isolates had SUL-DUR MIC values >4 µg/mL. Molecular characterization of these isolates confirmed the primary drivers of resistance are metallo-β-lactamases or penicillin-binding protein 3 (PBP3) mutations, as previously described. In addition, this study shows that certain common PBP3 variants, such as A515V, are insufficient to confer sulbactam resistance and that the efflux of durlobactam by AdeIJK is likely to play a role in a subset of strains.

## INTRODUCTION

Antimicrobial resistance has become a leading cause of death globally and is estimated to result in about 3,500 deaths per day (1). In addition, resistant Gram-negative infections are associated with higher hospital costs, length of hospital and ICU stay, and increased mortality (2). Of these, infections caused by *Acinetobacter* spp. remain one of the most important unmet medical needs (3). Globally, *Acinetobacter* is the fifth leading pathogen for deaths associated with antibiotic resistance (1). In the United States, infections caused by *Acinetobacter* spp. are ~2% of all Gram-negative healthcare-associated infections; however, they are associated with high morbidity and mortality due, in part, to high rates of multidrug and carbapenem resistance (4, 5). Notably, the high incidence of carbapenem-resistant *Acinetobacter* (CRAB) worldwide is due, in large part, to wide-spread acquisition of class D β-lactamases (5, 6).

Sulbactam-durlobactam (formerly sulbactam-ETX2514) (SUL-DUR) (Fig. S1) is a targeted β-lactam/β-lactamase inhibitor (BL/BLI) combination antibiotic that was designed to treat serious infections caused by *Acinetobacter*, including multidrug-resistant (MDR) and carbapenem-resistant strains, and has recently been approved by the FDA for treatment of hospital-acquired and ventilator-associated pneumonia (HABP/VABP) caused by susceptible isolates of *Acinetobacter baumannii-calcoaceticus* complex (ABC) (7, 8). Sulbactam is a β-lactam Ambler class A β-lactamase inhibitor (BLI) that also has intrinsic antibacterial activity against *Acinetobacter* spp., which is mediated through the inhibition of penicillin binding proteins (PBP1 and PBP3), essential components of cell wall synthesis (9 – 11). However, degradation of sulbactam by a variety of β-lactamases present in most *Acinetobacter* clinical isolates limits its clinical use (10, 12). Durlobactam is a non-β-lactam BLI which has a modified diazabicyclooctane (DBO) scaffold that inhibits a broad range of class A, C, and D β-lactamases (10, 13, 14). Durlobactam effectively restores sulbactam activity both *in vitro* and *in vivo* (10, 13, 14). The MIC_90_ of sulbactam alone against contemporary *Acinetobacter baumannii calcoaceticus* complex isolates in surveillance studies or in molecularly characterized collections of CRAB ranged from 32 to >64 µg/mL and was reduced to 2–8 µg/mL in the presence of durlobactam (14 – 18). The frequency of spontaneous resistance to SUL-DUR in diverse clinical isolates was 7.6 × 10^−10^ to <9.0 × 10^−10^ at 4× MIC (19). While most of these mutants mapped to PBP3, a few mapped to tRNA synthetase genes, *aspS* and *gltX*, which are associated with the stringent response (19). In addition, results from another study suggested that durlobactam could be a substrate for certain *Acinetobacter* efflux pumps, as efflux pump knockout strains (Δ*adeB*, Δ*adeJ*, and Δ*adeB*/Δ*adeJ*) were more susceptible to SUL-DUR but not sulbactam alone (13).

The most comprehensive surveillance study to date tested the *in vitro* activity of SUL-DUR against a collection of 5,032 ABC clinical isolates collected in 33 countries across the Asia/South Pacific region, Europe, Latin America, the Middle East, and North America from 2016 to 2021 (14). In this study, the addition of durlobactam (at a fixed concentration of 4 µg/mL) reduced the sulbactam MIC_50/90_ from 8/32 to 1/2 µg/mL. SUL-DUR activity was maintained across individual ABC species, years, global regions of collection, specimen sources, and resistance phenotypes, including MDR and extensively drug-resistant (XDR) isolates. Only 1.7% (84/5,032) of ABC isolates from 2016 to 2021 had SUL-DUR MIC values >4 µg/mL, its susceptibility breakpoint (20).

Upon retest in triplicate, 78 of these 84 were stably non-susceptible to SUL-DUR (MIC ≥8 µg/mL) (Table S1). The following report describes the genomic analysis of these isolates for likely resistance mechanisms. Biochemical analysis of purified PBP3 and phenotypic analyses of both clinical and isogenic strains were also conducted to determine the relative impact of key resistance determinants to SUL-DUR antibacterial activity.

## RESULTS

### Genetic characterization of isolates with reduced SUL-DUR susceptibility

**Fig 1:**
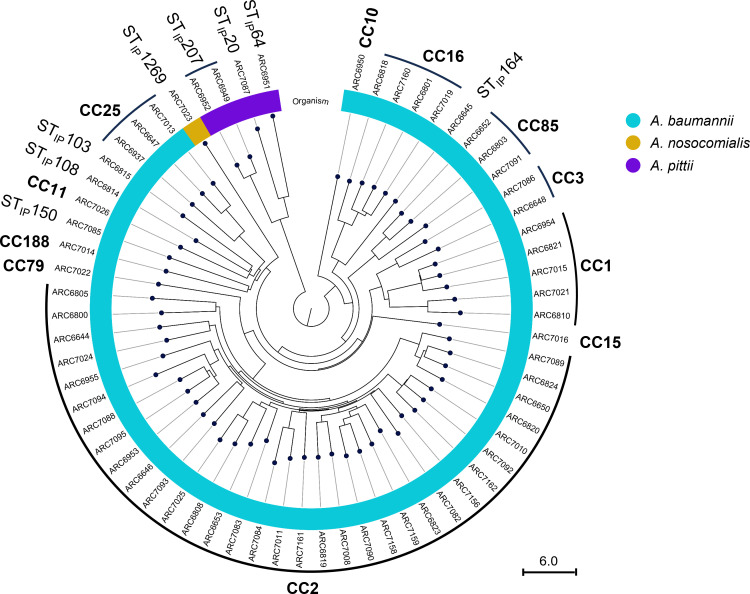
Circular phylogram tree of 64 SUL-DUR non-susceptible ABC isolates from a 6-year surveillance study (2016–2021). The clonal complex (CC) using Institut Pasteur scheme and Institut Pasteur MLST sequence types (ST_IP_) are indicated on the outer ring.

All 78 SUL-DUR non-susceptible isolates were carbapenem-resistant (Table S1). MLST and comparative genomic analyses revealed 14 of these isolates were clonal to at least one other isolate, resulting in 64 distinct genotypes, 33 (52%) of which belong to clonal complex 2 (CC2) using the Pasteur MLST scheme (Fig. 1; Table S3). This collection of isolates was highly antibiotic resistant (Table S2); in fact, 48 (75% of 64) were extensively drug resistant (21). Over half (58%, 33 of 64) had modest decreases in susceptibility to SUL-DUR (*N* = 21 and *N* = 12 with SUL-DUR MIC values of 8 and 16 µg/mL, respectively). Additional whole-genome sequencing analysis included the identification of β-lactamase genes, variations in PBPs, and efflux systems. Variants in several other factors previously detected in SUL-DUR-non-susceptible ABC isolates (whose role has yet to be defined) were also considered. This analysis revealed that 63 (98%) of the 64 isolates encoded previously defined SUL-DUR resistance elements, namely, either a PBP3 variant (*N* = 30), a metallo-β-lactamase (MBL) gene (*N* = 23), or both (*N* = 10) (Table S3).

### PBP variants

Of the 40 isolates found to encode for amino acid changes in PBP3, the most common mutant alleles were T526S (*N* = 19, five of which were in combination with an additional mutation at N377I/Y) and A515V (*N* = 12, one of which was in combination with N392T). Other PBP3 variants detected were K235N (*N* = 3), F548I (*N* = 2), G523V (*N* = 2, alone or in combination with T337I), A583V (*N* = 1), and V146I (*N* = 1) (Table S3). In addition, similar to a previous report (18), six isolates encoded mutations or transposon disruptions in either PBP1a or PBP1b, five encoded PBP2 variants, and five encoded PBP6b mutations (Table S3); however, all of these isolates also encoded either a PBP3 variant or an MBL gene. Therefore, the relative contribution of mutations in additional PBP genes to SUL-DUR susceptibility could not be defined based on genomic analysis alone. SUL-DUR MIC values for the isolates with PBP mutations that lacked MBL genes ranged from 8 to 64 µg/mL (Table S3).

### Sulbactam inhibition of purified PBP3 variants

**Fig 2:**
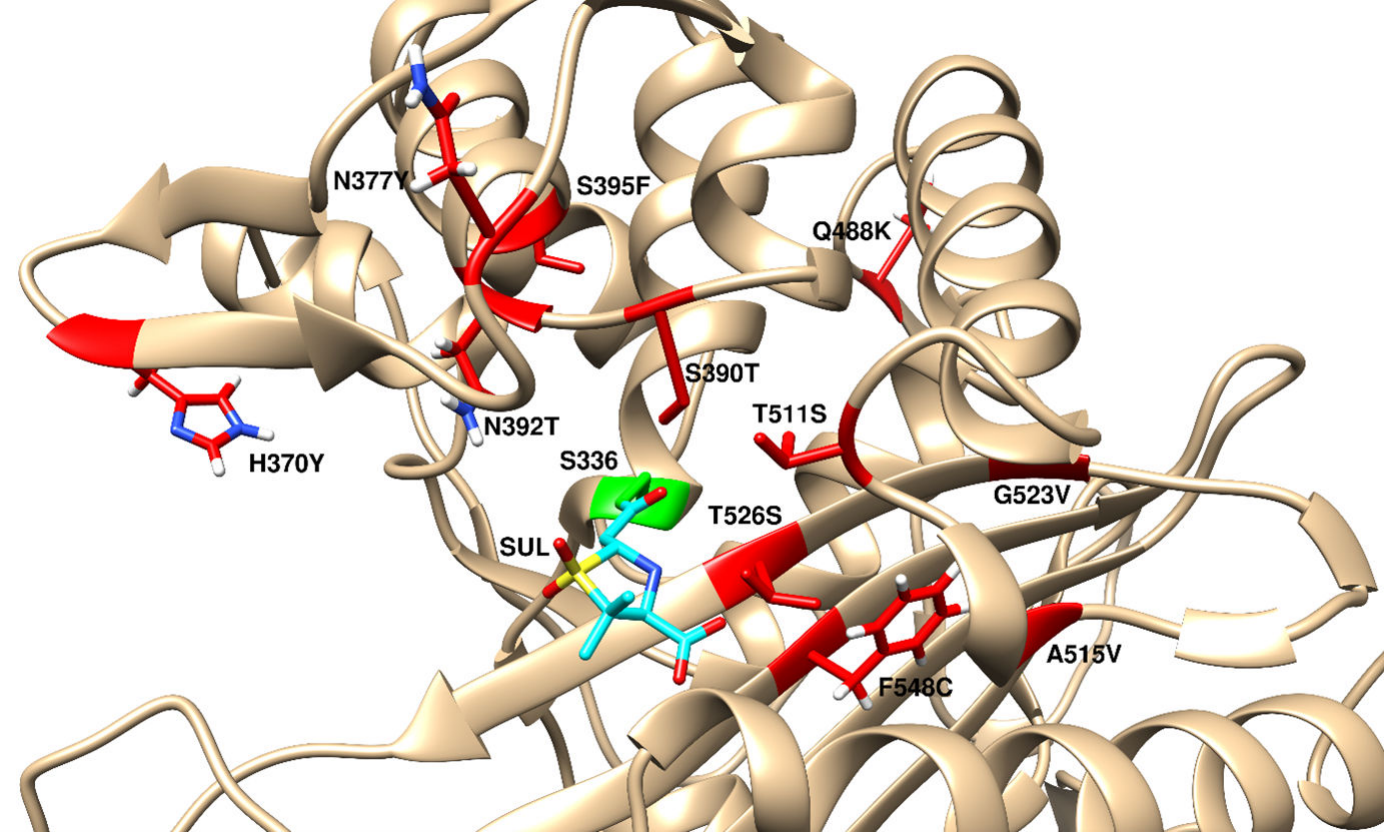
Structure of *A. baumannii* PBP3 (PDB ID 3ue3) with sulbactam (cyan) docked and covalently bound to the active site serine S336, shown in green. Variants detected in isolates with elevated SUL-DUR MIC values are indicated in red.

In a previous study, the acylation rate constant (*k*_inact_/*K*_i_) of sulbactam versus purified *A. baumannii* wild-type PBP3 was compared to S390T, S395F, and T511S variants, which had been generated in the laboratory in spontaneous resistance studies *in vitro* (19). All three of these variants had significantly reduced reactivity with sulbactam but varied in their ability to bind to comparator β-lactams (aztreonam, imipenem, or meropenem). To understand how the PBP3 variants identified in the 6-year surveillance study, as well as those described elsewhere, affect sulbactam reactivity, eight additional variants were cloned, expressed, and purified for characterization. A fluorescence anisotropy assay was used to measure the ability of sulbactam to compete with fluorescent BOCILLIN for time-dependent acylation of each PBP3 variant as compared to wild-type PBP3. PBP3 variants that demonstrated the greatest reduction in sulbactam acylation rate constants (≤25% of wild-type levels) were G523V, T526S, and F548C, similar to levels observed for S390T, S395F, and T511S. In contrast, A515V, N377Y, and Q488K had modest impacts on sulbactam acylation rate constants, ranging from 60%–73% of wild-type levels. Finally, N392T and H370Y PBP3 variants displayed slightly increased acylation rate constants compared to wild-type levels (~120%) (Table 1). The proximity of each of these variants to sulbactam bound to the active site of *A. baumannii* PBP3 is shown in Fig. 2. Notably, no correlation was found for the effect of the PBP3 variants on the acylation rate constants of meropenem, imipenem, aztreonam, and sulbactam, suggesting that all four beta-lactams have distinct binding modes (Table 1).

**TABLE 1:** Acylation rate constants (*k*_inact_/*K*_i_) (M^-1^s^-1^) for sulbactam, aztreonam, meropenem, and imipenem versus *A. baumannii* wild-type and mutant PBP3s

PBP3 variant	Sulbactam^*d*^		Meropenem		Imipenem		Aztreonam		
	*k*_inact_/*K*_i_	% WT	*k*_inact_/*K*_i_	% WT	k_inact_/K_i_	% WT	k_inact_/K_i_		% WT
Wildtype	15 ± 3 (10)	100	5,000 ± 1,000 (10)	100	450 ± 60 (10)	100	700 ± 100 (10)		100
H370Y^*a*^	19 ± 2 (2)	126.7	6,000 ± 10 (2)	120	480 ± 40 (2)	106.7	430 ± 30 (2)		61.4
N377Y^*b*^	11 ± 2 (2)	73.3	3,700 ± 300 (2)	74	280 ± 10 (2)	62.2	510 ± 60 (2)		72.9
S390T^*c*^	0.03 ± 0.02 (3)	0.2	16,000 ± 6,000 (3)	31.3	540 ± 40 (3)	120	900 ± 300 (3)		128.6
N392T^*b*^	18 ± 1 (2)	120	8,200 ± 200 (2)	164	790 ± 50 (2)	175.6	160 ± 20 (2)		22.9
S395F^*c*^	0.63 ± 0.06 (3)	4.2	1,500 ± 200 (3)	30	8.8 ± 0.7 (3)	2.0	110 ± 20 (3)		15.7
Q488K^*b*^	9.9 ± 0.3 (2)	66	3,900 ± 200 (2)	78	370 ± 20 (2)	82.2	460 ± 50 (2)		65.7
T511S^*c*^	1.45 ± 0.09 (3)	9.7	3,400 ± 400 (3)	68	220 ± 20 (3)	48.9	450 ± 80 (3)		64.3
A515V^*b*^	9 ± 2 (4)	60	1,405 ± 7 (2)	28.1	120 ± 0 (2)	26.7	590 ± 40 (2)		84.3
G523V^*b*^	0.938 ± 0 (2)	6.3	1,000 ± 100 (2)	20	89 ± 2 (2)	19.8	440 ± 20 (2)		62.9
T526S^*b*^	0.7 ± 0.1 (4)	4.7	1,300 ± 100 (2)	26	38 ± 2 (2)	8.4	900 ± 90 (2)		128.6
F548C^*b*^	1.1 ± 0.3 (3)	0.7	3,000 ± 600 (3)	60	140 ± 20 (3)	31.1	500 ± 100 (3)		71.4

^*a*^ Barnes et al. (2019).

^*b*^ This study.

^*c*^ McLeod et al. (2018).

^*d*^ Values shown are the average ± standard deviation (*n*). WT, wildtype.

### Effect of PBP3 variants on SUL-DUR activity in an isogenic *A. baylyi* panel alone or in the presence of overexpressed β-lactamases

To understand the effect of PBP3 variants on whole cell activity in the absence of other resistance determinants, the MIC values of sulbactam, SUL-DUR and comparator β-lactams were measured against an isogenic *A. baylyi* panel engineered to contain individual strains in which the endogenous PBP3 was replaced with the variant of interest. The panel contained the two most common PBP3 variants associated with decreased SUL-DUR susceptibility in the 6 y surveillance study: T526S and A515V, which showed 95% and 40% reduction in sulbactam acylation rate constants as compared to wildtype PBP3, respectively. In addition, the G523V and H370Y PBP3 variants, which showed 94% and 126% sulbactam acylation rate constants relative to wildtype, respectively, were also engineered and tested. As shown in Table 2, A515V, H370Y, and G523V PBP3 variants had minimal effects on susceptibility to all the agents tested as compared to wildtype PBP3, but expression of the T526S PBP3 variant resulted in an 8-fold increase in both sulbactam and SUL-DUR MIC values. However, it did not affect carbapenem or aztreonam activity.

**TABLE 2:** MIC values (µg/mL) of sulbactam, SUL-DUR and comparator β-lactams against an isogenic *A. baylyi* panel encoding wildtype or mutant PBP3 alone or in the presence of five different overexpressed β-lactamases

overexpressed β-lactamase	Compound	PBP3 variant				
		Wildtype	H370Y	A515V	G523V	T526S
None (empty vector)	sulbactam	4	8	4	8	32
	SUL-DUR	1	1	1	2	8
	imipenem	0.03	0.03	0.06	0.13	0.13
	meropenem	0.06	0.06	0.13	0.25	0.06
	aztreonam	32	64	32	32	32
TEM-1	sulbactam	16	4	16	16	>64
	SUL-DUR	1	1	1	2	16
	imipenem	0.03	0.03	0.03	0.13	0.06
	meropenem	0.13	0.06	0.06	0.25	0.03
	aztreonam	32	64	32	32	32
ADC-30	sulbactam	2	2	4	8	64
	SUL-DUR	2	1	1	2	8
	imipenem	0.13	0.03	0.06	0.25	0.13
	meropenem	0.13	0.06	0.13	0.25	0.13
	aztreonam	32	32	32	32	32
OXA-23	sulbactam	>64	>64	32	32	>64
	SUL-DUR	2	2	1	4	8
	imipenem	8	4	2	8	>8
	meropenem	8	8	4	8	>8
	aztreonam	64	64	32	32	32
OXA-24	sulbactam	16	>64	8	32	>64
	SUL-DUR	1	2	2	4	32
	imipenem	8	>8	>8	>8	>8
	meropenem	>8	>8	>8	>8	>8
	aztreonam	32	64	32	32	32
OXA-66	sulbactam	8	4	4	8	32
	SUL-DUR	1	1	1	4	16
	imipenem	0.13	0.06	0.13	0.5	0.5
	meropenem	1	0.5	0.5	2	1
	aztreonam	32	64	32	32	32

### β-Lactamase-mediated resistance

Each of the *A. baylyi* panel members were then transformed with plasmids that constitutively expressed five distinct, representative β-lactamases commonly found in *Acinetobacter* clinical isolates (TEM-1, ADC-30, OXA-23, OXA-24 or OXA-66) and evaluated for changes in antibiotic susceptibility. For the strain encoding wildtype PBP3, sulbactam MIC values increased ≥4 fold in the presence of TEM-1, OXA-23 and OXA-24 but not ADC-30 or OXA-66. Imipenem MIC values increased ≥4 fold in the presence of ADC-30, OXA-23, OXA-24, and OXA-66 while meropenem MIC values increased ≥4 fold in the presence of OXA-23, OXA-24 and OXA-66. As predicted, no or small (≤2 fold) changes in MIC values were observed for either SUL-DUR (due to the inhibition of β-lactamases by durlobactam), or aztreonam (which is not a substrate for any of these enzymes) (22, 23). Similar (≤2 fold difference) MICs in sulbactam and SUL-DUR were observed for strains expressing A515V, H370Y, and G523V PBP3 as for those with wildtype PBP3. For strains with the T526S background, sulbactam MIC values shifted from 32 µg/mL in the absence of a β-lactamase to >64 µg/mL in the presence of TEM-1, OXA-23 and OXA-24. SUL-DUR MIC values shifted from 8 µg/mL to 16–32 µg/mL in the presence of all enzymes tested except ADC-30 and OXA-23 (Table 2). Taken together, these results suggest the T526S PBP3 variant confers *bona fide* resistance to SUL-DUR, which is exacerbated in the presence of overexpressed β-lactamases, while the A515V PBP3 variant had little to no effect on SUL-DUR activity in whole cells, regardless of the presence or type of β-lactamase expression.

The majority of SUL-DUR nonsusceptible isolates that encoded A515V PBP3 also encoded for NDM-1 (Table S3). Notably, of the 33 MBL-positive *Acinetobacter* isolates, 32 encoded *bla*_NDM-1_ and one encoded *bla*_VIM-4_; 27 of these had SUL-DUR MIC values of ≥32 µg/mL. Because durlobactam does not inhibit metallo-β-lactamases (10), weak SUL-DUR activity is expected against these isolates.

The contribution of serine β-lactamase expression to decreased SUL-DUR activity in isolates that encoded PBP3 A515V but were devoid of MBL genes was evaluated by comparing the rate of sulbactam hydrolysis in whole cell extracts with or without pretreatment with 4 µg/mL durlobactam. Sulbactam was fully protected from hydrolysis by durlobactam in all isolates tested. In addition, there was no correlation between SUL-DUR MIC value and relative sulbactam hydrolytic activity (Fig. S2).

### Efflux variants

Overexpression of three different RND efflux systems (AdeABC, AdeFGH and AdeIJK) have been associated with multidrug resistance in *A. baumannii* (24). Of the 64 SUL-DUR nonsusceptible ABC isolates described above, 11 (17%) had no detectable genetic changes in efflux systems; 21 (33%) lacked *adeC*, the outer membrane component of one of the RND efflux systems in *Acinetobacter,* and the entire *adeABCRS* operon was missing in two isolates (Table S3). *adeC* is reported to be absent in ~40% of clinical isolates. It has been shown to be nonessential for the efflux function (25, 26), and its function can be complemented by AdeK (27), so the *adeC* null mutants identified in this study are unlikely to contribute to altered SUL-DUR susceptibility. Additional variants in components of the AdeABC, AdeFGH, AdeIJK or AdeT RND efflux systems were detected in 47 (73% of 64) of these isolates. Notably, six isolates encoded mutations in the AdeRS two-component regulatory system (which controls expression of the AdeABC efflux system), two isolates encoded mutations in AdeL (repressor of the AdeFGH efflux system), and 19 isolates encoded mutations in AdeN, the repressor of the AdeIJK efflux system. Mutations in all three repressors have been shown to result in an increase in the efflux of certain antibiotics (27). However, all but one isolate with mutations in efflux systems also encoded for PBP3 variants and/or for MBLs, making it difficult to define the extent to which efflux affects SUL-DUR activity in these isolates based on genotype alone.

To further assess the role of efflux in SUL-DUR activity against *Acinetobacter* spp., two panels of *A. baumannii* isogenic strains were tested. The first consisted of 7 members of an *A. baumannii* transposon library (28) with single gene disruptions in the *adeABC*, *adeIJK*, or *adeT* loci as compared to the parent strain, AB5075. The second collection consisted of 11 genetically engineered strains in the KAB1544 background, each of which resulted in overexpression of one of three efflux systems. This panel of strains consists of four different point mutants in AdeR and five different point mutants in AdeS, which result in the upregulation of AdeABC expression. In addition, this panel contains chromosomal deletions of *adeL* and *adeN*, which result in overexpression of AdeFGH and AdeIJK, respectively (29).

The parent and each isogenic mutant strain in both panels was measured for susceptibility to sulbactam, durlobactam, SUL-DUR, imipenem, and gentamicin. In the transposon library with single gene disruptions, AdeB and AdeA disruptions resulted in gentamicin MIC shifts of >8 to >64 fold as compared to the parent strain, respectively, as expected since gentamicin has been previously shown to be an efflux substrate for the AdeABC efflux system (30) (Table 3). Minimal changes in MIC values as compared to the parent strain were seen for sulbactam and imipenem, with only 2-fold MIC changes for all tested efflux gene disruptions. However, 4-fold changes in MIC values as compared to the parent strain were observed for the *adeJ*::Tn and *adeK*::Tn mutants for durlobactam alone as well as SUL-DUR, the former of which has been previously described (13). Minimal changes in MIC for durlobactam alone or SUL-DUR were observed for the AdeABC and AdeT gene disruptions (Table 3).

**TABLE 3:** *In vitro* activity of sulbactam and durlobactam alone or in combination and comparator compounds against isogenic *A. baumannii* transposon mutants in efflux genes^*a*^

Isolate ID	Sulbactam	Durlobactam	SUL-DUR	IPM	GEN
Parent (AB5075)	64	128	2	16	>64
*adeA::tn*	32	64	2	8	1
*adeB::tn*	32	256	2	8	8
*adeC::tn*	64	256	2	16	>64
*adeI::tn*	32	64	1	16	>64
*adeJ::tn*	64	32	0.5	16	>64
*adeK::tn*	32	32	1	16	>64
*adeT::tn*	64	128	2	8	>64

^*a*^ Each agent was tested in duplicate.

For the efflux overexpression panel, most of the AdeR and AdeS mutants resulted in an 8- to 16-fold increase in gentamicin MIC values relative to the parent strain, as expected (30). In contrast, sulbactam, SUL-DUR, and imipenem MIC values were unaffected by these mutants (Table 4). For the Δ*adeL* and Δ*adeN* deletion mutants, minimal changes in MIC values were observed for any of the compounds tested. Taken together, results from both isogenic panels suggest that the absence of a functional AdeIJK efflux system, as shown by the efflux pump *adeJ::tn* isolate, has a greater effect on SUL-DUR antibacterial activity than its overexpression.

**TABLE 4:** *In vitro* activity of sulbactam and durlobactam, alone or in combination, and comparator compounds against isogenic *A. baumannii* overexpression efflux mutants^*a*^

		MIC (µg/mL)				
Efflux pump overexpressed	Strain	Sulbactam	Durlobactam	SUL-DUR	Imipenem	Gentamicin
None	Parent (KAB1544)	2	128	0.5	0.25	2
AdeABC	AdeR D20N	1	64	0.5	0.5	16
	AdeR A91V	1	32	0.25	0.5	32
	AdeR P116L	1	64	0.5	0.5	16
	AdeR E219A	2	128	0.5	0.25	2
AdeABC	AdeS G30D	1	64	0.25	0.5	32
	AdeS A94V	1	64	0.5	0.5	16
	AdeS G103D	2	64	0.5	0.25	2
	AdeS R152K	1	64	0.5	0.5	16
	AdeS T153M	1	32	0.25	0.5	32
AdeFGH	Δ*adeL*	1	64	0.5	0.25	2
AdeIJK	Δ*adeN*	2	128	1	0.25	2

^*a*^ Each agent was tested in duplicate.

**Fig 3:**
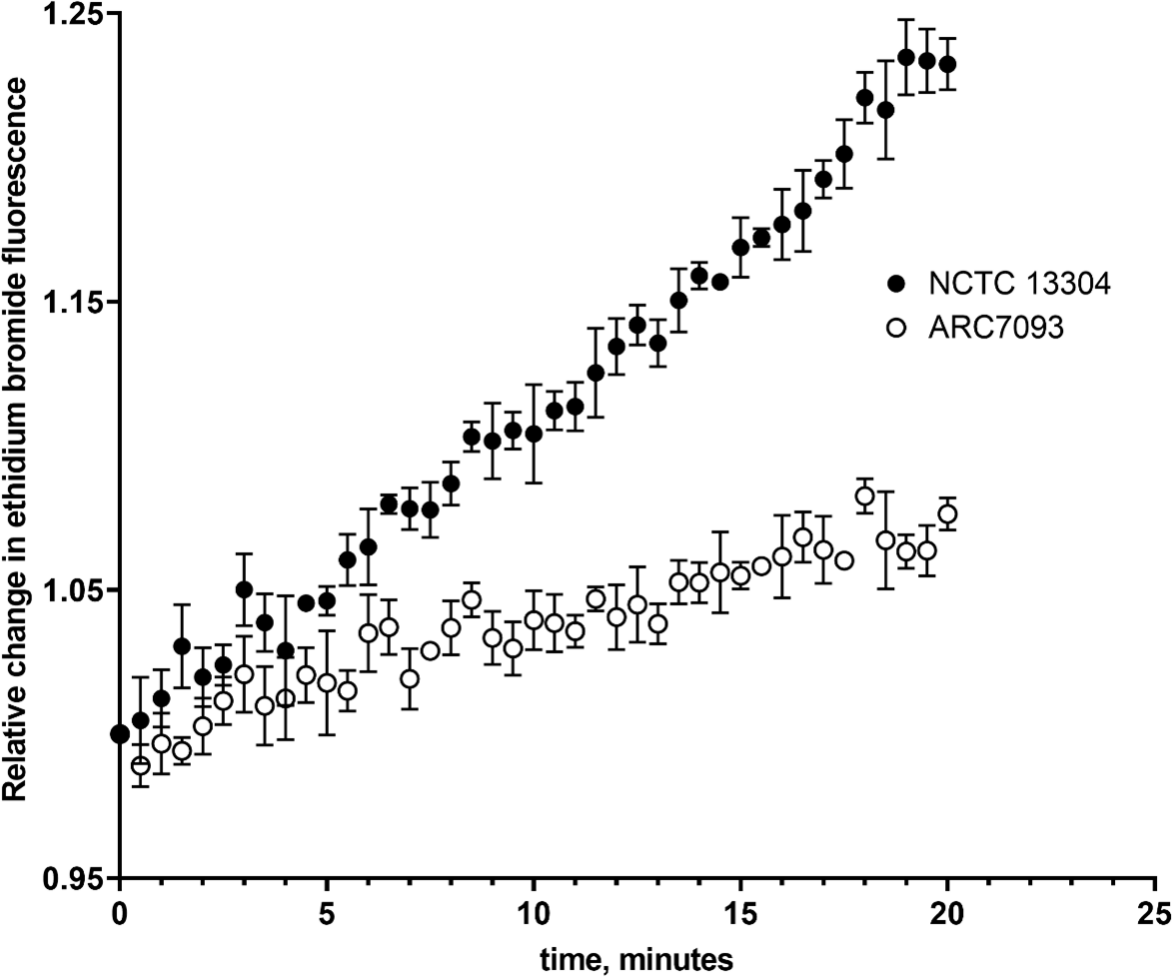
Inherent efflux potential of NCTC 13304 and ARC7093, which encodes AdeJ [G288S]. The rate of accumulation of ethidium bromide is plotted as a relative change in accumulation on the Y-axis. A significant difference in ethidium bromide accumulation was found between these strains using an unpaired *t*-test, *P* < 0.0001. The *X*-axis represents time in minutes. Error bars represent standard deviation around the mean and values are from three replicates.

Notably, ARC7093, the only SUL-DUR non-susceptible isolate (out of 64) that did not encode for either a PBP3 variant or an MBL gene, was found to encode a missense mutation in AdeJ (G288S) (Table S3). This AdeJ variant has been previously described in the context of carbapenem resistance due to the overexpression of the AdeJ pump, although the association was only made based on genotype, not phenotype (31). The efflux capacity of ARC7093 was, therefore, compared to NCTC 13304, a SUL-DUR-susceptible clinical isolate with wild-type AdeIJK, in an ethidium bromide accumulation assay. The accumulation of ethidium bromide in ARC7093 over 20 min was significantly lower (*P* < 0.0001) than in NCTC 13304, suggesting that ARC7093 has a higher efflux capacity (Fig. 3). Therefore, the AdeJ (G288S) variant may play a role in susceptibility to SUL-DUR in *Acinetobacter* spp.

### Additional elements

Only one of the isolates with reduced SUL-DUR susceptibility (ARC6821) was found to encode any variation in porin genes: a single frameshift mutation in *carO*, which has been associated with carbapenem resistance (5). However, this frameshift mutation in *carO* was in combination with a PBP3 [T526S] mutation (strain ARC6821, Table S3), so the contribution of *carO* to SUL-DUR non-susceptibility cannot be definitively determined. Notably, none of the isolates with elevated SUL-DUR MIC values had mutations in *ompA*, the gene encoding for the essential, multifunctional outer membrane porin OmpA, which both sulbactam and durlobactam use to permeate the outer membrane *of A. baumannii* (32). Therefore, altered porin expression does not appear to be a major driver of decreased susceptibility to SUL-DUR in *Acinetobacter* spp. (32).

Mutations in genes encoding for other factors encoded by either sulbactam- or SUL-DUR-resistant isolates have been reported on rare occasions. These include variants in *aspRS, bfmRS, galE, gltX, ldtJ, ldtK, mraY, rpoC,* and *spoT* (9, 19). Twenty-one (33%) of isolates in this study encoded at least one mutation in *bmfRS, galE, gltX, spoT, aspS,* or *rpoC* genes (Table S3). Because these variants appeared inconsistently across strains and have also been detected in SUL-DUR susceptible strains (data not shown), their role in SUL-DUR activity is likely either minimal or strain-dependent.

## DISCUSSION

The studies described here both confirm and extend previous observations related to the molecular drivers of resistance to SUL-DUR in contemporary isolates of ABC. Nearly all (63 of 64) of the ABC isolates with SUL-DUR MIC values >4 µg/mL from a 6-year surveillance study encoded metallo-β-lactamases, which durlobactam does not inhibit (10), and/or mutations in PBP3 that reduce sulbactam binding affinity (19). There was a notable difference in relative SUL-DUR susceptibility for these drivers of resistance: nearly all MBL-expressing ABC isolates had SUL-DUR MIC values ≥32 µg/mL, whereas SUL-DUR MIC values for the majority of PBP3 variants were 8 or 16 µg/mL (Table S3). The relative effect of each PBP3 variants on sulbactam potency was evaluated both biochemically and in the context of whole cell activity in genetically engineered *Acinetobacter* isogenic panels. Of the variants tested in the latter system, only the T526S allele of PBP3 resulted in significantly decreased sulbactam activity, which decreased further in the presence of a subset of clinically relevant β-lactamases (Table 2). Addition of durlobactam improved but did not fully restore sulbactam activity in these strains. In contrast, the G523V PBP3 variant demonstrated decreased susceptibility (≥4-fold difference) to both sulbactam and SUL-DUR only in the presence of OXA-23 or OXA-24 expression (Table 2). Of particular interest was the finding that the second most common PBP3 variant, A515V, was equally susceptible to sulbactam as the wildtype comparator, even in the presence of β-lactamase overexpression. This variant showed only a 40% reduction in sulbactam acylation rate constant compared to wild-type PBP3 (Table 1), which suggests that this mutation is insufficient to confer sulbactam non-susceptibility in whole cells. Therefore, additional factors likely play a role in SUL-DUR resistance in isolates that encode the PBP3 A515V variant. Notably, this variant showed a much greater effect on carbapenem acylation rate constant (<30% of wildtype levels, Table 1). Therefore, it is possible the PBP3 A515V variant is enriched in clinical isolates due to prior carbapenem exposure.

Results from an isogenic efflux transposon mutant panel suggest that durlobactam is a substrate for the AdeIJK efflux system (Table 3), consistent with previous reports (13). This hypothesis is supported by the observation that the single SUL-DUR-non-susceptible surveillance isolate in this study that did not encode an MBL or PBP mutation, ARC7093, had a G288S mutation in AdeJ and showed increased efflux capacity as compared to a SUL-DUR-susceptible strain with a wild-type AdeJ (Fig. 3). Notably, in a recent compassionate use case, a burn patient who was infected with pan-drug-resistant *A. baumannii* that encoded both PBP3 H370Y and AdeJ G288S experienced clinical cure after SUL-DUR therapy in combination with other antibiotics (33). Thirty-three percent of isolates characterized in this study were missing the outer membrane component of the AdeABC efflux system *adeC*, which is consistent with other studies, including the observation that AdeC has been shown to be non-essential for the efflux function as other outer membrane proteins can complement its function (25 – 27), so its effect on SUL-DUR resistance is likely negligible.

To date, no evidence exists to suggest porins are involved in SUL-DUR resistance, including *carO,* which has been linked to carbapenem resistance (34), and *ompA* , through which both SUL and DUR permeate (32). The contribution of other elements, such as mutations in other PBPs, genes involved in stringent response, or other genes with missense mutations identified in Table S3, remain to be defined. While previous *in vitro* resistance studies with SUL-DUR identified missense mutations in the stringent response in isolates with elevated MICs (19), such mutations in non-susceptible clinical isolates identified and genotyped in this study were always found in combination with either PBP3 missense mutants or in the presence of NDM-1 (Table S3).

Recently, SUL-DUR was approved by the FDA and is indicated in patients 18 years of age and older for the treatment of HABP/VABP, caused by susceptible isolates of *Acinetobacter baumannii-calcoaceticus* complex (8). Results from this and other studies suggest that, while resistance to SUL-DUR in clinical isolates exists, primarily due to either the presence of NDM-1 β-lactamase or certain mutations in PBP3, these types of *A. baumannii* isolates currently remain rare (14).

## MATERIALS AND METHODS

### Bacterial isolates

The 5,032 ABC isolates used for surveillance studies were collected by clinical laboratories in 33 countries from 2016 to 2021, as previously described (14). The 84 isolates with MICs >4 µg/mL were shipped to Entasis Therapeutics Inc. for additional testing, including repeat antimicrobial susceptibility and whole-genome sequencing.

The isogenic *A. baylyi* panel used for chromosomal expression of various PBP3 mutants with or without constitutive expression of various β-lactamases were constructed as part of this study. The *A. baylyi* ADP1 PBP3 ORF was cloned into pUC19 with a gentamicin cassette inserted downstream of the PBP3 ORF for selection purposes. Additionally, 500 bp of flanking homology to the ADP1 genome were inserted upstream of the PBP3 ORF and downstream of the gentamicin cassette. PBP3 variants (H370Y, A515V, G523V, and T526S) were introduced into this plasmid by PCR using primers that encoded for the variants. Linear DNA consisting of the 500 bp upstream-PBP3-Gent-500 bp downstream fragment was cloned out of the pUC19 plasmid by PCR and used for transformation of the ADP1 strain. The strain was grown at 30°C overnight in LB medium shaking at 250 rpm. Seventy microliters of the overnight ADP1 culture were combined with 1 mL of fresh LB and 200 ng of linear DNA fragment and incubated for 5 h at 30°C and shaking at 200 rpm. Various dilutions of each culture were plated on LB agar supplemented with 10 µg/mL of gentamicin to select for transformants with successful chromosomal integrations. Integrants were verified by Sanger sequencing using purified genomic DNA.

For the constitutive expression of various β-lactamases, ORFs of *bla*_TEM-1_, *bla*_ADC-30_, *bla*_OXA-23_, *bla*_OXA-24_, and *bla*_OXA-66_ were cloned by PCR from various clinical strains that encode these genes and inserted into the pWH1266 plasmid modified to encode a kanamycin cassette for selection. These plasmids along with an empty vector were used to transform each of the *A. baylyi* ADP1 strains engineered above that encode wild-type PBP3 or variants. Transformations were carried out as done above with selection on LB agar supplemented with 20 µg/mL of kanamycin.

The AB5075 parent strain as well as transposon interrupted AdeABC, AdeIJK, and AdeT were purchased from the Manoil laboratory at the University of Washington from their arrayed transposon mutant collection (28). The KAB1544 parent as well as efflux pump regulator mutants and deletions were constructed as previously described (29).

### Antimicrobial susceptibility testing

Antimicrobial susceptibility testing was performed using broth microdilution following standardized methods established by the Clinical Laboratory Standards Institute (CLSI) (35, 36). Quality control testing was performed as specified by CLSI using *A. baumannii* NCTC 13304 (35). MIC values were interpreted using CLSI breakpoints for all antimicrobial agents except those for which CLSI breakpoints are not available (35). SUL-DUR was tested as twofold dilutions of sulbactam in combination with a fixed concentration of 4 µg/mL durlobactam (35).

### Whole-genome sequencing and analysis

Extraction of chromosomal DNA, whole-genome sequencing, and subsequent analysis of genomic content of each isolate with a SUL-DUR MIC >4 µg/mL were performed at Entasis Therapeutics Inc. Chromosomal DNA was extracted from each isolate using the Promega Maxwell 16 instrument and Maxwell 16 Cell DNA Purification kit following the manufacturer’s protocol (Promega, Madison, WI). DNA was quantified with a Qubit 2.0 fluorometer using the dsDNA Broad Range Assay kit (Life Technologies, Grand Island, NY). DNA was diluted to 0.2 ng/µL, and 2.5 µL was used for library generation using the Nextera XT DNA sample preparation kit and Nextera XT index primers (Illumina, San Diego, CA). The recommended procedure was followed except the library normalization step was omitted in favor of qPCR library quantification. qPCR was performed on a BioRad CFX96 cycler using the NEBNext Library Quant Kit for Illumina (Ipswich, MA). Libraries were diluted to a standard concentration of 4 nM of DNA, and 2.5 µL of each sample (8–12 samples, targeted 25- to 50-fold coverage) was combined and denatured with 0.1 N NaOH (final) for 5 min. The sample was diluted to 600 µL to provide a 15–20 pM multiplex library. Samples were sequenced on an Illumina MiSeq instrument using the V2 chemistry in a 2 × 150 paired-end read format.

Assembly and analysis of whole-genome sequencing were performed using CLC Genomics Workbench v22.0 (Qiagen). Fastq files were processed and analyzed as follows: duplicate sequence reads were removed, and remaining reads were trimmed for quality and minimum length (50 bp). Reads were *de novo* assembled at high stringency (fraction length = 0.9, similarity fraction = 0.99) using default mismatch/insertion/deletion costs.

Strains with reduced susceptibility to SUL-DUR were selected for sequence analysis of cell wall synthesis, efflux, porin, as well as other genes of interest. The corresponding amino acid sequences were compared with the reference sequence of *A. baumannii* strain ATCC 17978 (Genbank accession number CP000521 .1). The resultant variations in the amino acids of the proteins are listed in Table S3. The β-lactamase content of each strain was determined by BLAST within the CLC Genomics Workbench against an assembled database of genes curated at Entasis Therapeutics Inc., with sequences originating from the NCBI Bacterial Antimicrobial Resistance Reference Gene Database (accession number PRJNA313047). For MLST determination, assembled contigs were exported from CLC Genomics Workbench and uploaded into the PubMLST database (https://pubmlst.org/databases/). For *Acinetobacter*, PubMLST hosts two different MLST schemes, Oxford and Institute Pasteur. The Oxford scheme (ST_ox_) assigns sequence types using the following genes: *gltA, gyrB, gdhB, recA, cpn60, gpi,* and *rpoD*. Alternatively, the Institute Pasteur scheme (ST_IP_) assigns sequence types using alleles of *cpn60, fusA, gltA, pyrG, recA, rplB,* and *rpoB*. Sequence types from both schemes are reported when available. Clonal complex (CC) for each strain was determined using the Pasteur scheme by grouping strains with single locus variants (37). CCs are noted in Table S3; Fig. 1; singleton strains not grouped in a specific CC are noted with identified ST_IP_ in Fig. 1. The circular phylogram was constructed in CLC Genomics v22.0 using the whole-genome alignment tool, with minimum initial seed length = 15, followed by the creation of average nucleotide identity comparison with minimum similarity fraction = 0.8 and minimum length fraction = 0.8. The circular tree was created from the comparison by neighbor joining.

Strains collected from the same clinical site in the same year with identical MIC and MLST profiles were tested for genetic clonality. Clonality of strains was determined in CLC Genomics Workbench v22.0 by first *de novo* mapping one of the strains of interest (as discussed above) and then using that genome as a reference for mapping raw reads of the second strain of interest. Raw reads were trimmed for quality and minimum length, overlapping pairs were merged, and then reads were mapped to the *de novo* assembled reference strain with length fraction = 0.7 and similarity fraction = 0.8. Variants were determined by the basic variant detection tool in CLC Genomics, with a minimum coverage requirement of 7×, minimum count of 2, and minimum frequency of 70%. Automatic detection of variants was followed up with manual inspection of reads. Strains with less than five single-nucleotide polymorphisms in total were classified as clonal.

### Purification of PBP3 variants and determination of acylation rate constants

DNA manipulation, cloning, expression, purification, and determination of acylation rate constants of *A. baumannii* PBP3 variants in this study against sulbactam, meropenem, imipenem, and aztreonam were performed as previously described (38). Briefly, the *A. baumannii* PBP3 gene-encoding residues 64–609 fused to a C-terminal hexahistidine tag was cloned into pET28a. Variants constructed in this study were introduced by PCR using primers encoding the nucleotide changes. Once the sequence was verified by Sanger sequencing, BL21(DE3) cells were transformed with the variant PBP3 encoding pET28a plasmids and inoculated into 1 L of LB medium supplemented with 25 µg/mL kanamycin. Cultures were grown at 37°C and shaken at 250 rpm to an OD_600_ ~0.6, cooled on ice for 30 min, and induced with 0.01 mM IPTG overnight at 16°C and shaking at 250 rpm. After 16 h, cells were harvested and frozen at −80°C. Cell paste from each culture expressing the variant PBP3 was resuspended in 35 mL of buffer containing 25 mM Tris-HCl (pH 8.0), 400 mM NaCl, 10 mM imidazole, 10% (v/v) glycerol, and 0.1% CHAPS detergent and passed through a French press at 4°C twice at 18,000 psi. The extract was centrifuged at 8,260 *× g* for 30 min at 4°C. The supernatant was applied to a 5-mL HisTrap Ni column (Cytiva) equilibrated with the same buffer at a flow rate of 2 mL/min. The column was washed with the same buffer until the 280 nm absorbance returned to baseline and then eluted with a 10 column-volume linear gradient of 0.01–0.5 M imidazole in the same buffer. Fractions containing PBP3, based on SDS-PAGE, were pooled and concentrated using Amicon Ultra-15 centrifugal ultrafiltration units. The concentrated protein was applied to a 120-mL HiLoad 16/60 Superdex 200 column (Cytiva) equilibrated with 25 mM Tris-HCl (pH 8.0), 200 mM NaCl, and 10% (v/v) glycerol at 1 mL/min. Fractions containing PBP3 were pooled, concentrated using Amicon Ultra-15 centrifugal ultrafiltration units, and stored at −80°C. Acylation rate constants for each variant tested against sulbactam and comparators were determined as previously described (38).

### Protection of SUL by DUR in whole-cell extracts

The protection of sulbactam by durlobactam in the extracts of strains of interest was performed as follows. Overnight cultures were diluted 1:100 in 25 mL of MHBII medium and incubated with shaking at 35°C until the OD_600_ reached 0.6–1. Cells were collected by centrifugation at 5,000 × *g* for 10 min at 4°C. Cell pellets were resuspended in 1 mL of ice-cold 0.1 M sodium phosphate (pH 7.0), and centrifuged at 5,000 × *g* for 5 min at 4°C. Cell pellets were resuspended in 0.3 mL of extraction buffer [0.1 M sodium phosphate (pH 7.0), 1 mM EDTA, 1× HALT EDTA-free protease inhibitor cocktail (Thermo-Fisher Scientific)]. Cells were lysed by five freeze-thaw cycles. Unlysed cells and cell debris were pelleted by centrifugation at 16,873 × *g* for 10 min at 4°C. Protein concentrations of the supernatants were measured by Bradford assay (Bio-Rad Laboratories). Each strain extract was diluted 1:5 in 0.1 M sodium phosphate (pH 7.0), 10 mM sodium bicarbonate, and 0.005% Triton X-100 detergent, and 22.5 µL of each were aliquoted into UV-transparent 384-well plates in triplicate. For each strain, 22.5 µL of either buffer or 200 µM sulbactam were added to each extract in the 384-well plate, and the absorbance at 235 nm was read at 20 s intervals for 1 h on a Spectramax Plus 384 plate reader (Molecular Devices). The average absorbance for each strain from wells with buffer only was subtracted from those of the sulbactam-containing wells, and initial rates of Δ*A*_235_ versus time progress curves starting at 10 min were calculated. For the protection of sulbactam degradation by β-lactamases present in the extracts, the extracts were preincubated with 4 µg/mL durlobactam for 1 h.

### Ethidium bromide uptake assay

NCTC 13304 and ARC7093 were cultured overnight on Tryptic Soy Blood Agar plates. Single colonies were used to inoculate 2 mL of Mueller-Hinton Cation-adjusted Broth (MHBII) and grown overnight at 35°C with shaking. On the day of the experiment, the overnight culture was diluted 100-fold into a flask containing 20 mL of MHBII and grown under the same conditions to OD_600_ ~0.2. Two-mL aliquots of this logarithmic phase culture were centrifuged. The pellets were gently re-suspended in 2-mL of sterile phosphate-buffered saline (pH 7.0) containing 8 µM EtBr. The cells were transferred to a black-wall, clear-bottom 96-well microplate, and EtBr fluorescence (excitation—530 nm, emission—590 nm) was measured over a period of 20 min in a SpectraMax M5e plate reader (Molecular Devices). Statistical significance for the accumulation of ethidium bromide was determined using an unpaired *t*-test.

## Data Availability

All genome sequencing data described in this manuscript have been deposited at DDBJ/ENA/Genbank as part of Bioproject PRJNA988092, under the accession JAUEMN000000000 – JAUEOY000000000. The versions described in this paper are versions JAUEMN010000000 – JAUEOY010000000.

## SUPPLEMENTAL MATERIAL

Supplemental Figures S1 and S2 aac.00665-23-s0001.docx Document containing Supplemental Figures S1 and S2.

Supplemental Tables S1 to S3 aac.00665-23-s0002.xlsx Excel sheet with supplemental tables S1 to S3, each in a separate tab.

ASM does not own the copyrights to Supplemental Material that may be linked to, or accessed through, an article. The authors have granted ASM a non-exclusive, world-wide license to publish the Supplemental Material files. Please contact the corresponding author directly for reuse.
