# Supplementary material for: Molecular drivers of resistance to sulbactam-durlobactam in contemporary clinical isolates of Acinetobacter baumannii
Source: Antimicrob Agents Chemother. 2023 Oct 16;67(11):e00665-23. doi: 10.1128/aac.00665-23 (PMC10648852; doi:10.1128/aac.00665-23)
Supplement: Supplemental Figures S1 and S2 — Document containing Supplemental Figures S1 and S2. [file aac.00665-23-s0001.docx]

**Supplemental Figure S1.** Chemical structures of sulbactam (left) and durlobactam (right).

**
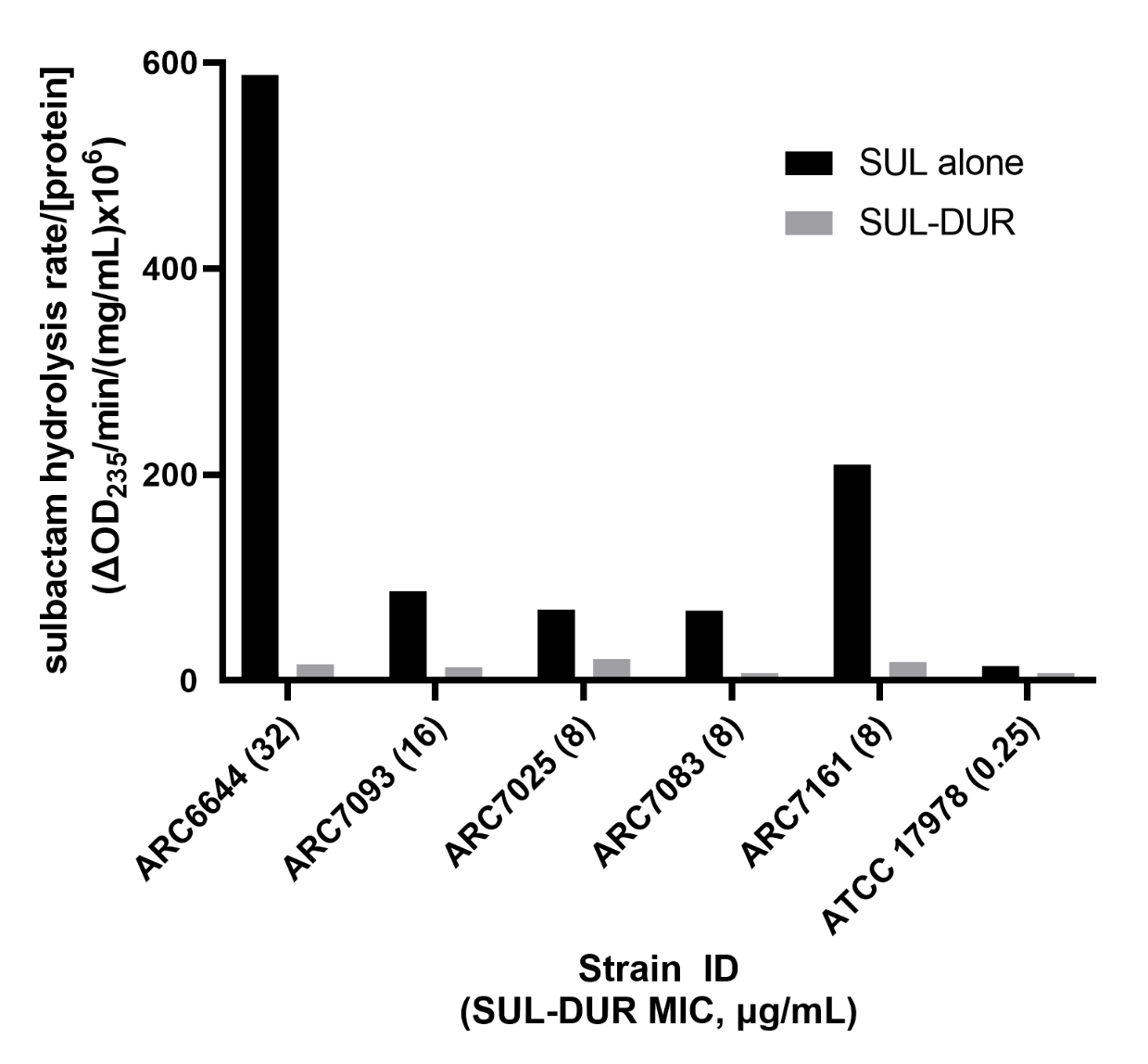
**

**Supplemental Figure S2.** Sulbactam hydrolysis rate in whole cell extracts of selected SUL-DUR non-susceptible isolates alone or in the presence of 4 µg/mL durlobactam. SUL = sulbactam; DUR = durlobactam.
